# Potential risks associated with Fe, Zn, Cu, Ni, and Cr contamination in the muscle of *Sardina pilchardus* (Walbaum, 1792) from Morocco

**DOI:** 10.1039/d5ra05445a

**Published:** 2025-11-20

**Authors:** Khaoula Kasmi, Hanae Nasri, Douaa Slimani, Rajae Mouedden, Souad Abdellaoui, Kamal Belhaj, Abdelhafid Chafi

**Affiliations:** a LAPABE, Faculty of Sciences, University Mohammed First Mohammed VI Street, P.O. Box 524 Oujda 60000 Morocco kasmikhaoula77@gmail.com; b LSAM, Higher School of Technology Sidi Bennour, University Chouaib Doukkali Jabran Khalil Jabran Street, P.O. Box 299-24000 El Jadida Morocco belhaj.kamal@ucd.ac.ma

## Abstract

Marine fish face increasing threats from human activities, particularly pollution, which exacerbates marine contamination. This study evaluates the risks of trace element contamination in the edible muscle of Sardina pilchardus from Morocco. Using inductively coupled plasma optical emission spectrometry (ICP-OES), concentrations of iron (Fe), zinc (Zn), Copper (Cu), nickel (Ni), and chromium (Cr) were quantified in 360 specimens. Zinc was the most abundant metal (70.28 µg g^−1^ dry weight (dw)), followed by Fe (60.31 µg g^−1^ dw), Cu (2.31 µg g^−1^ dw), and Ni (0.83 µg g^−1^ dw), while Cr was undetected. Seasonal variations significantly influenced metal concentrations and health risk indices (*p* < 0.05), with higher Fe and Zn levels in colder seasons and elevated Ni and Cu levels during warmer periods. No significant differences were found between sampling locations (*p* > 0.05). All recorded levels were below international food safety standards. Health risk assessments, including estimated daily intake, target hazard quotient, and total THQ, indicated no immediate health risk. However, toxicological evaluation revealed that the target carcinogenic risk (CR) for Ni exceeded the threshold of 10^−4^, suggesting a potential long-term carcinogenic risk. While trace element levels in *Sardina pilchardus* generally pose no immediate health concerns, the elevated CR for Ni emphasizes the need for further research, monitoring, and mitigation strategies. These findings underline the importance of continuous assessment to ensure the safety of marine fish and to mitigate potential risks to human health over time.

## Abstract 2

Marine fish face increasing threats from human activities, particularly pollution, which contributes to the worsening of marine contamination.

## Graphical abstract

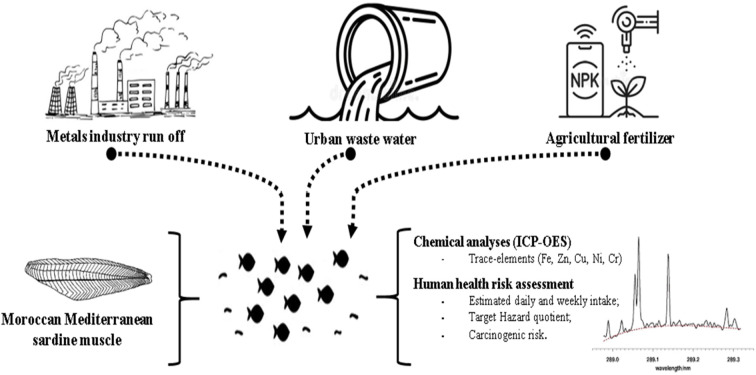

## 1. Introduction

Daily, hundreds of pollutants are released into the environment, particularly the aquatic ecosystems that are more susceptible to heavy metal pollution especially with the increasing development of industrial activities and the excessive use of pesticides in agriculture. Among these contaminants, oligo-elements are significant pollutants in aquatic ecosystems because they persist over time and tend to accumulate in the tissues of aquatic organisms.^1^ Trace elements are present in small quantities in the body, but have very important biological functions.^2^ Despite their infinitesimal doses, they can be highly toxic if overdosed. Exposure to these toxic metals can happen through different pathways such as inhaling contaminated air, consuming polluted food, or direct skin contact with polluted water or air.^3^ The Mediterranean Sea stretches approximately 3.860 kilometers in length and covers an area of about 2.5 million square kilometers, making it the largest semi-enclosed sea in the world. Its connection to the Atlantic Ocean through the Strait of Gibraltar is only 14.3 kilometers wide at its narrowest point. This makes it a hotspot for biodiversity. Although it serves as an economic lifeline for many countries, it is also one of the most polluted seas. The sardine is considered one of the most consumed fish species due to its significant nutritional value, being a valuable source of essential nutrients such as high-quality proteins, essential amino acids, and omega-3 fatty acids (docosahexaenoic acid and eicosapentaenoic acid) vitamins such as vitamin D, B12 and minerals namely iron, magnesium and calcium.^4^ Also, its economic affordability, ecological abundance and integration into local culinary traditions makes it a significant edible fish. The Mediterranean sardine (*Sardina pilchardus*, Walbaum, 1792) is a staple in the Moroccan diet and a crucial component of the country's fishing industry. With increasing concerns about marine pollution and its impact on seafood safety, assessing the levels of heavy and trace metals contamination in sardine flesh has become imperative. In light of these considerations, this study aims to evaluate the concentration of Iron (Fe), Zinc (Zn), Copper (Cu), and Nickel (Ni) in sardines caught off the Moroccan Mediterranean coast. Understanding these contamination levels is essential for ensuring public health and maintaining the integrity of marine ecosystems.

## 2. Experimental

### 2.1. Sampling

**Fig. 1:**
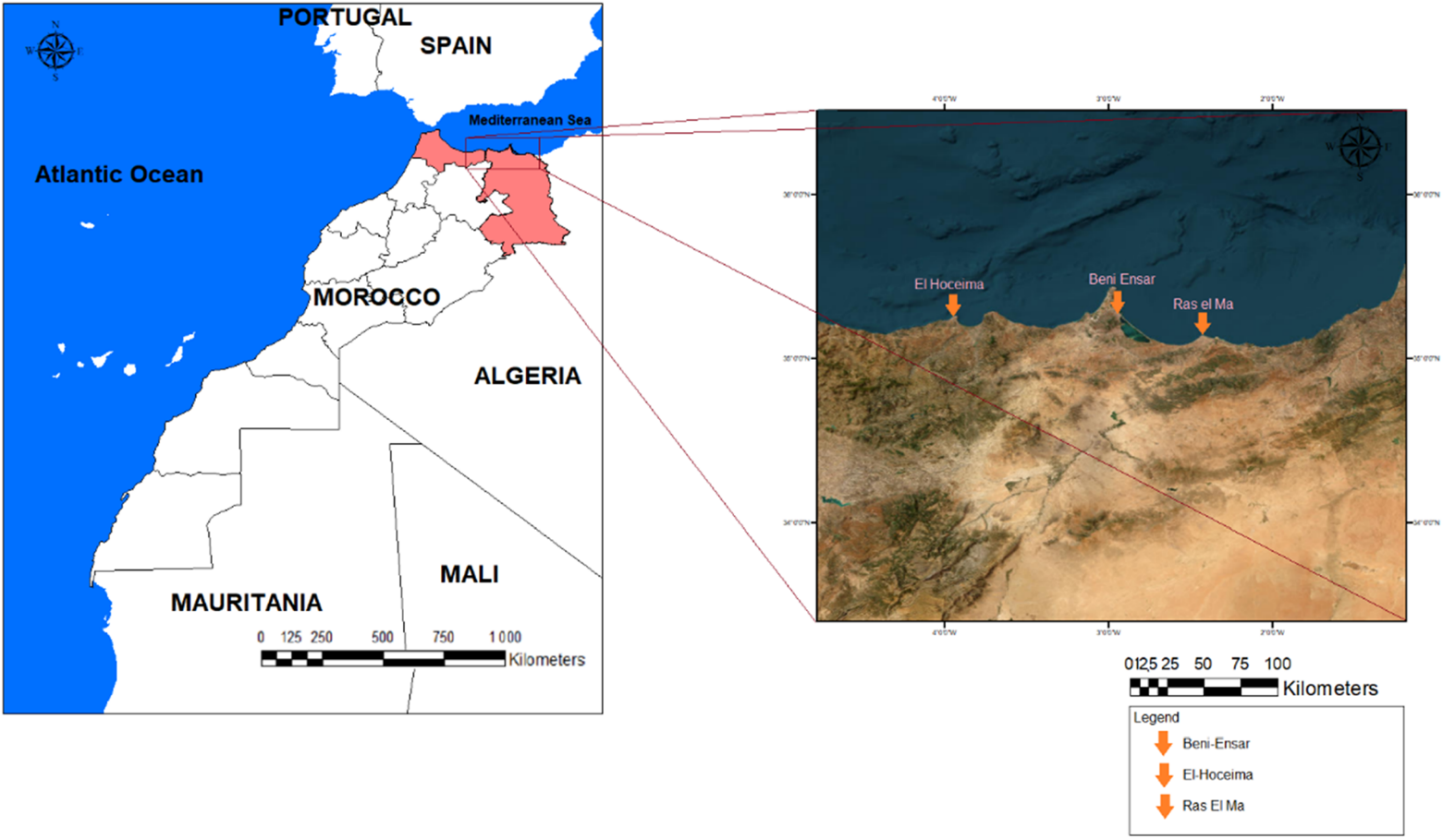
The three study sites along the Moroccan Mediterranean coast.

Over four seasons, from December 2020 to November 2021, a total of three hundred sixty sardines were collected from three sites along the Mediterranean coast of Morocco, using artisanal fishing nets. The sampling followed a 4 × 3 factorial design, representing the seasons and areas. Every season, 10 specimens were collected monthly, resulting in a total of thirty samples for the entire season. The specimens were categorized into four groups for each area, with thirty specimens per area for each season. *Sardina pilchardus* (*S. pilchardus*) were collected from three ports along a 512 km stretch of the Mediterranean coastline: El-Houceima Sea, Beni-Ensar Sea, and Ras el Ma Sea (see Fig. 1).

### 2.2. Samples preparation

In the laboratory, the Moroccan Mediterranean sardine muscles were first rinsed using distilled water followed with dissection with stainless steel scissors. The muscle samples were dried and ground into a fine powder using an agate mortar to avoid metal contamination, and stored under vacuum in sterilized pouches at −18 °C for further analysis. To prevent any potential contamination, acid-cleaned laboratory equipment was used throughout both the preparation and during analysis.

### 2.3. Chemical analyses

For the chemical analysis of trace element concentrations (Fe, Cu, Zn, Ni, and Cr), 1 to 2 grams of dried muscle tissue were used. The samples digestion and preparation were described in our published article. Metal contents were analyzed using inductively coupled plasma optical emission spectrometry (ICP-OES, PerkinElmer Optima 5300 DV). The results are presented in µg g^−1^ dry weight. The moisture content was estimated using the oven drying method at 100 °C ± 3 °C.^5^

### 2.4. Human health risk assessment

The content values were utilized to estimate the potential human health risks associated with exposure to metals, considering both carcinogenic and non-carcinogenic effects among Moroccan consumers. The assessment included the estimated daily intake (EDI: eqn (1)), target hazard quotient (THQ: eqn (2)), total hazard quotient index (TTHQ: eqn (3)), and carcinogenic risk (CR: eqn (4)). These indices were not estimated for metals with concentrations below the ICP-OES detection limit. In the absence of official data on sardine-specific consumption in Morocco, the average national sardine intake was estimated based on the total Moroccan population (35 586 616 inhabitants),^6^ the national sardine production reported by the same institution, and the quantity of sardines exported according to the Sea Report in Figures.^7^

The resulting estimate corresponds to an average consumption of 14.23 kg of sardines per capita per year, compared to the overall national fish consumption of 19.47 kg per capita per year.^8^ This estimation was used to calculate the EDI and THQ values in the present study.

#### 2.4.1. Estimated daily intake (EDI)

The EDI was estimated using eqn (1), considering the concentrations of the studied trace metals in sardine edible muscle and the average daily fish consumption in Morocco.1
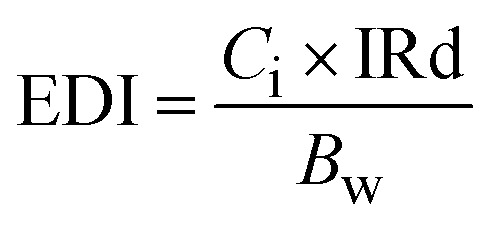
where: EDI is presented in µg g^−1^ wet weight; *C*_i_ represents the metals concentration in fish (µg g^−1^ ww); he weekly ingestion rate (IRd), expressed in grams per day, is estimated at 38 g per person per day^6^ and the term *B*_w_ refers to body weight, which in Morocco averages 73.35 kg for adults.^9^

#### 2.4.2. Non-carcinogenic risk indices

##### 2.4.2.1. Target hazard quotient (THQ)

The THQ for non-carcinogenic health risk is calculated as the ratio of contaminant exposure to the oral reference dose (RfD). If the calculated indices are below 1, this indicates that consuming sardines from the Mediterranean coast of Morocco is generally safe concerning these metals. Conversely, a THQ value of 1 or higher suggests a potential health risk, indicating that exposure may exceed safe levels, particularly with frequent consumption or high metal concentrations. The THQ was calculated using the following eqn (2):2
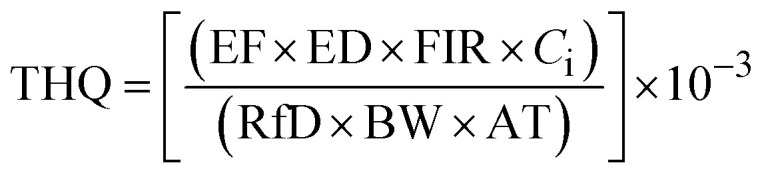
where: The exposure frequency (EF) is considered to be 365 days per year, indicating continuous daily exposure. The average lifetime duration (ED) for the Moroccan population is 76.9 years.^10^ The fish ingestion rate (FIR) is estimated at 38 g/person/day.^7,11^ The metal concentration in the fish sample is denoted as *C*_i_. The RfD for trace elements is given in µg g^−1^ day^−1^, with values of 700 for Fe, 300 for Cu, 40 Zn, and 20 for Ni. The average BW of an adult consumer is 73.35 kg for the Moroccan population.^9^ The mean time of exposure to non-carcinogens (AT) is calculated as 365 days per year multiplied by ED.

##### 2.4.2.2. Total hazard quotient index (TTHQ)

The TTHQ represents the total health risk resulting from exposure to multiple contaminants, highlighting the overall potential risk associated with the presence of more than one metal. It is determined by calculating the total of the target hazard quotients (THQs) for each metal. The THQ was calculated using the following eqn (3):

3 TTHQ = THQ_(Iron)_ + THQ_(Zinc)_ + THQ_(Copper)_ + THQ_(Nickel)_ + THQ_(Chromium)_

where TTHQ value below 1 indicates no risk associated with consuming sardine muscle, while a TTHQ value above 1 suggests a potential risk for intake of Fe, Zn, Cu, Ni, and Cr.

#### 2.4.3. Carcinogenic risk index

Carcinogenic risk (CR) is used to estimate the probability of an individual developing cancer over their lifetime due to exposure to potential carcinogens. The CR was calculated using the eqn (4):4
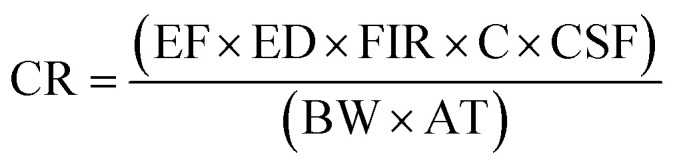
where: the carcinogenic slope factor (CSF) refers to the oral CSF provided by the Integrated Risk Information System (µg g^−1^ day^−1^). Since Fe, Zn, and Cu do not have carcinogenic effects, the carcinogenic risk (CR) was assessed only for Ni and Cr. The oral carcinogenic slope factors (CSFo) are 0.5 µg g^−1^ day^−1^ for Ni and 0.91 µg g^−1^ day^−1^ for Cr.^12–14^ The EF, ED, FIR, C, BW, and AT are already explained above. If the CR value is less than 10^−6^, it is considered negligible; a CR value between 10^−6^ and 10^−4^ is deemed acceptable; while a CR above 10^−4^ is regarded as unacceptable.

### 2.5. Quality control and assurance

The analysis of the certified reference material, DORM-4 fish protein, obtained from the National Research Council of Canada, validated both precision and quality control. The limits of detection and limits of quantification were as follows: 0.06 and 0.12 µg g^−1^ for Fe, 0.04 and 0.13 µg g^−1^ for Zn, 0.04 and 0.12 µg g^−1^ for Cu, 0.02 and 0.04 µg g^−1^ for Ni, and 0.01 µg g^−1^ for Cr. The recovery rates ranged from 98% to 104% for all trace elements studied.

### 2.6. Statistical analysis

IBM SPSS was used for statistical analyses. A two-way analysis of variance (ANOVA) was carried out with a 5% significance level to assess whether there were significant differences in metal concentrations among different sites and seasons. The Shapiro–Wilk test was utilized to evaluate the normality of the data. Tukey's *post hoc* test was employed for mean comparisons when significant differences were detected (*p* < 0.05). Additionally, chemometric analysis was applied to the dataset to distinguish the results based on sampling time (seasons) and geographical area (site).

## 3. Results and discussion

### 3.1. Metals, season and area variation

Trace elements are naturally present in small concentrations in both the environment and the human body. They are essential for the proper functioning of various biological systems, and maintaining their levels within recommended ranges is crucial for optimal physiological balance.^2^ In fish flesh, metals such as Fe, Zn, Cu, Ni, and Cr are absorbed and play key roles in numerous physiological processes. However, when these metals exceed the recommended levels set by regulatory authorities, they are classified as ‘toxic metals’ due to their harmful effects on both the fish and its consumers.^15^ These metallic elements are known for their high toxicological impact and persistence in the environment. They can bioaccumulate and biomagnify through the food chain, resulting in negative combined effects on human health and disrupting the balance of aquatic ecosystems.^16^ Fish can absorb and accumulate toxic metals from their aquatic habitats through various pathways, including ingestion, ion exchange through the gills, or adsorption onto tissue surfaces.^17^ Humans consume fish extensively for their nutritional benefits, as they are rich in essential amino acids, PUFAs n-3 and PUFAs n-6 fatty acids, balanced omega 3/omega6 ratio, and provide essential minerals and vitamins.^18^ Due to their harmful effects on both fish and human health, the accumulation of metals in fish gills, muscles, and livers has garnered increased attention. This study assesses the levels of trace metals in the muscle tissue of *S. pilchardus* collected from three Moroccan Mediterranean ports across the four seasons. The focus is on muscle tissue since it is the most commonly consumed part of the fish, despite not being the primary site for metal accumulation.^16^Tables 1 and 2 show the effect of geographical area and season on the contamination levels of Fe, Zn, Cu, and Ni in the muscle of *S. pilchardus* from the Mediterranean coast of Morocco. The analyze of variance (ANOVA) show no significant variation based on geographical area (*p* > 0.05). In contrast, the trace elements analyzed were significantly affected by both the season (*p* < 0.05) and the interaction season*site (*p* < 0.001). Cr was not detected in any of the analyzed samples. The most prevalent metals ions in all specimens were Zn in winter season, and Fe came in the second order (Table 1). Generally, the contents of the studied trace elements were in the following order: Zn (70.28 µg g^−1^ dry weight (dw)) > Fe (60.31 µg g^−1^ dw) > Cu (2.31 µg g^−1^ dw) > Ni (0.83 µg g^−1^ dw) > Cr (not detected). In the seasonal variation 2 increased orders were observed for three studied Seas (Table 2). In the winter season, the concentration of metals in muscle samples followed the order: Zn > Fe > Cu > Ni. However, during the spring, the sequence shifted to: Fe > Zn > Cu > Ni. Among the trace metals, Nickel recorded the lowest levels across all locations and seasons. The statistical analysis demonstrates a significant season effect (*p* < 0.05). For Fe and Zn, the high values were recorded in cold seasons (winter and spring) (*p* < 0.001, Table 2). Similar effect were reported in the literature by several authors.^13,19^ This seasonal variation in metals levels is likely due to a complex interplay of environmental factors, dietary changes, reproductive cycles, anthropogenic activities, and biological processes. Understanding these processes requires further research, particularly to distinguish the relative contributions of each factor to the observed variations. Concerning Cu and Ni the greatest values are recorded in hot seasons (summer and autumn) (*p* < 0.001, Table 2). This difference could be explained by the high temperatures of summer which can increase the metabolic rates of fish, potentially leading to higher Cu uptake. Additionally, changes in water currents and upwelling during different seasons can bring Cu-rich waters to the surface, increasing the exposure of fish to Cu. Furthermore, increased human activities, particularly agriculture and tourism, during the summer could lead to higher Cu levels in coastal waters. Similarly, increased boat traffic and industrial activities in summer could contribute to elevated Cu levels in the water.

**Table 1:** Mean metal contents, along with their SD, for Fe, Zn, Cu, and Ni in the sardine muscle from the Moroccan Mediterranean coasts, categorized by season^a^

Metals (µg g^−1^ dw)	Area	Wn	Sp	Sm	Aut
Iron	Beni Ensar	81.81^c^ ± 5.34	59.10^b^ ± 11.06	39.90^a^ ± 1.07	54.49^b^ ± 4.80
	Ras el Ma	85.78^c^ ± 0.24	76.69^c^ ± 2.26	41.44^a^ ± 6.79	52.59^b^ ± 1.97
	El Houceima	85.73^c^ ± 1.16	54.59^b^ ± 0.60	43.08^a^ ± 0.66	59.86^b^ ± 0.76
Zinc	Beni-Ensar	103.41^c^ ± 14.15	63.93^b^ ± 2.95	43.70^a^ ± 3.89	73.52^b^ ± 3.42
	Ras el Ma	129.90^c^ ± 1.44	56.59^a^ ± 5.35	50.14^a^ ± 4.04	69.64^b^ ± 1.90
	El Houceima	121.21^c^ ± 1.07	45.05^b^ ± 1.15	52.44^b^ ± 2.08	33.82^a^ ± 0.55
Copper	Beni-Ensar	3.14^b^ ± 0.53	2.41^a^ ± 0.01	3.17^b^ ± 0.52	3.90^b^ ± 0.1
	Ras el Ma	1.33^a^ ± 0.01	2.36^b^ ± 0.05	1.53^a^ ± 0.01	1.25^a^ ± 0.08
	El Houceima	<0.01^a^ ± 0.000	2.83^c^ ± 0.04	4.36^d^ ± 0.02	1.39^b^ ± 0.01
Nickel	Beni-Ensar	<0.01^a^ ± 0.000	<0.01^a^ ± 0.000	1.01^b^ ± 0.001	1.26^c^ ± 0.02
	Ras el Ma	<0.01^a^ ± 0.000	1.37^c^ ± 0.05	1.05^b^ ± 0.06	1.76^c^ ± 0.01
	El Houceima	0.47^b^ ± 0.001	<0.01^a^ ± 0.000	1.39^c^ ± 0.01	1.18^c^ ± 0.01
Chromium	Beni-Ensar	<0.01^a^ ± 0.000	<0.01^a^ ± 0.000	<0.01^a^ ± 0.000	<0.01^a^ ± 0.000
	Ras el Ma	<0.01^a^ ± 0.000	<0.01^a^ ± 0.000	<0.01^a^ ± 0.000	<0.01^a^ ± 0.000
	El Houceima	<0.01^a^ ± 0.000	<0.01^a^ ± 0.000	<0.01^a^ ± 0.000	<0.01^a^ ± 0.000

a The results are presented in µg g^−1^ dry weight (dw); significant differences (*p* < 0.05) between means are indicated by different lowercase letters (a–d); SD: standard deviation; Wn: winter; Sp: spring; Sm: summer; Aut: autumn.

**Table 2:** Variance analysis of the effects of site, season, and their interaction on contamination levels of Fe, Zn, Cu, and Ni in the sardine muscle from the Moroccan Mediterranean coasts

	Degree of freedom	Mean squares	*F*	*P*
**Iron**				
Site	3	1.406	0.265	>0.05
Season	2	2876.342	140.644	<0.001
Site × season	6	56.673	2.771	<0.05
Error	24	20.451		

**Copper**				
Site	3	7.265	7.748	<0.01
Season	2	3.700	3.946	<0.05
Site × season	6	4.33	4.624	<0.05
Error	24	0.938		

**Zinc**				
Site	3	548.365	10.085	<0.01
Season	2	4083.951	75.111	<0.001
Site × season	6	3215.899	59.146	<0.001
Error	24	54.372		

**Nickel**				
Site	3	1.075	23.113	<0.001
Season	2	3.362	72.261	<0.001
Site × season	6	0.498	10.698	<0.001
Error	24	0.047		

The concentration of Fe ranged from 39.90 µg g^−1^ dw in summer for specimens from Beni-Ensar to 85.78 µg g^−1^ dw in winter for specimens from the Ras el Ma Sea. Fe is an essential nutrient, playing a crucial role in oxygen transport and cellular respiration.^20^ The presence of Fe at these levels suggests that while sardines contribute beneficial Fe to the diet in particular the heme form.^21,22^ The recorded Fe levels were within acceptable limits set by international food safety standards (700 mg g^−1^ wet weight (ww)),^13,23^ indicating that these concentrations do not pose a significant health risk. The recorded seasonal variations are in agreement with those reported by several studies.^13,19^ On other hand, the recorded values are lower than those reported by,^19^ and comparable to those reported by^24^ while are higher than those reported by^13^ and in muscle of sardine (Table 3). These findings could be attributed to natural or anthropogenic contamination. Additionally, understanding the differences between studies is challenging due to the unclear geographical origins of the samples and potential variations in fish diet, predation habits, and species-specific characteristics. It is suggested that variations in trace element content are not only due to contaminant exposure but also to the natural accumulation processes within marine organisms, influenced by a range of biological and environmental factors.

**Table 3:** Comparison of metals accumulation in the muscle of sardine flesh and canned fish^a^

Site	Metals (µg g^−1^ wet weight)					Reference
	Fe	Zn	Cu	Ni	Cr	
Beni-Ensar, Morocco	8.50–17.43	9.31–22.03	0.51–0.84	0.002–0.27	ND	Current study
Ras el Ma, Morocco	8.91–18.43	10.78–27.93	0.29–0.51	0.002–0.38	ND	Current study
El-Houceima, Morocco	9.05–18.00	7.10–25.45	0.002–0.92	0.002–0.29	ND	Current study
Todos os Santos Bay, Brazil	4.61–99.39	4.11–34.99	0.23	—	0.04–0.22	33
Mediterranean, Spain	—	2.972–12.281	0.237–1.081	—	—	34
Yellow sea, China	6.62–25.2	4.22–36.6	0.193–0.493	0.048–0.209	0.089–0.493	35
Mediterranean, Egypt	3.88–4.10	0.88–0.93	0.05–0.15	0.32–0.48	0.10–0.22	13
Indian ocean, Bangladesh	—	24.66	3.61	ND	ND	14
Aegean sea, Turkey	50.54–68.22	44.11–74.18	0.03–0.05	ND	ND	19
Atlantic ocean, Morocco	6.5	8	0.6	0.25		24
PTDI (in µg kg^−1^ body weight per day)	700	300	40	20	3	36
RDA (mg day^−1^)	11	11	0.9	N/A	N/A	34 and 37

a The results are presented as mean; ND: not detected; Fe: iron; Zn: zinc: Zn; Cu: copper; Ni: nickel; Cr: chromium; PTDI: permissible tolerable daily intake in µg kg^−1^ body weight per day;^36^ RDA: Recommended Dietary Allowance; N/A: not available; The conversion factors used to convert dry weight to wet weight are 0.210, 0.213, and 0.215 for Beni-Ensar, Ras el Ma, and El-Houceima, respectively.

Regarding the Zn, the recorded values in the sardine flesh ranged between 33.82 µg g^−1^ dw in summer for El-Houceima samples and 129.90 33.82 µg g^−1^ dw in winter for those of Ras el Ma Sea. These values were also found to be within permissible limits (300 mg g^−1^).^23^ Zn is vital for numerous biological functions, including immune response and enzyme activity.^25^ The detected levels align with the nutritional importance of sardines as a source of Zn.^19^ report the comparable results to our in turkey, meanwhile^13^ and^24^ reported a lower values (Table 3). The observed variation in contamination levels can be attributed to differences in geographical areas, availability of feed, water quality, and anthropogenic contamination. These factors impact the solubility, bioavailability, and distribution of metals, which in turn affect the physiological condition of the aquatic system's biota.^26^

The values registered for Cu varied from 0.01 µg g^−1^ dw in winter to 4.36 µg g^−1^ dw in summer for El-Houceima specimens muscle. The recorded levels were generally low than permissible value (40 mg g^−1^ ww), indicating minimal contamination.^23^ Cu is vital for numerous bodily functions, such as the red blood cell production and the maintenance of healthy nerves and immune function.^27^ The low levels found suggest that there is no immediate risk of Cu toxicity from consuming these fish. This variability in Cu concentrations across different regions could be due to varying environmental factors or anthropogenic activities. The higher values were recorded in the El Houceima and Beni-Ensar regions during the hot seasons, which are known for high population density and intense tourism activity in the summer, potentially explaining the differences observed in Cu levels. Our results exceed those reported by^13,24^ and^19^ in muscle of Sardina from Egypt, Morocco and Turkey, respectively (Table 3). In contrast, the recorded values for cu levels are lower than those reported by^28^ in fish muscles from the Marmara, Aegean, and Mediterranean seas.

Concerning nickel (Ni), the detected values ranged between 0.01 µg g^−1^ dw in winter and spring for samples from Beni-Ensar Sea and 1.76 µg g^−1^ dw in summer for El Houceima specimens. The concentrations were lower compared to other metals. Prolonged exposure to elevated levels of Ni can lead to adverse health effects, including respiratory problems and allergic reactions.^29,30^ The recorded values in the edible tissues of the studied specimens do not exceed the WHO's regulatory limit for Ni in fish muscle, which is set at 20 mg g^−1^ww. The higher values are recorded in hot season highlighting the importance of monitoring nickel levels regularly to ensure they do not reach harmful concentrations especially in hot seasons. For winter season, our findings are below those documented by Monier *et al.*^13^ (2023), similar in spring, but higher in summer and autumn (Table 3). In contrast, are lower than those observed by Türkmen *et al.*^28^ (2008) in sardine muscle from the Marmara, Aegean, and Mediterranean seas. Finally, the chromium was undetected in all analyzed sardine muscle samples, confirming the absence of detectable chromium contamination in the study area.

The observed seasonal variations in trace element concentrations in sardine muscle may reflect corresponding changes in seawater chemistry. Several studies have reported that the concentrations of Fe and Zn tend to increase in colder seasons due to upwelling and enhanced nutrient input, while Cu and Ni often rise during warmer periods as a result of increased anthropogenic activity and stratification of the water column.^31–33^ These environmental fluctuations likely contribute to the bioaccumulation patterns observed in the present study.

From a nutritional perspective, considering the Recommended Dietary Allowance (RDA) for each element (see Table 3), the exanimated Mediterranean sardine muscle provides only a small percentage of the RDA for the studied essential elements. Even Zn and Fe, which are present at higher concentrations compared to other elements, contribute only about 1% of the RDA. In summary, the sardine muscle offers a modest contribution to the RDA for Fe (heme iron), Zn, and Cu, ranging from approximately 0.14% for Cuto 1.18% for Zn, Table 3.

### 3.2. Risk health assessment

Evaluating the THQ and TTHQ for sardine muscle provides valuable insight into the potential non-carcinogenic health risks for consumers. This health risk assessment was conducted using both non-carcinogenic and carcinogenic indices for Fe, Zn, Cu, and Ni in *S. pilchardus* from the Mediterranean coast of Morocco. The evaluation included metrics such as EDI, THQ, TTHQ, and CR. Tables 4 and 5 presents the health risk indices calculated for each metal, site, and season. As indicated, the calculated values for EDI, THQ, and TTHQ show no statistically significant difference (ANOVA; *p* > 0.05, Table 5) based on sampling location, though they are influenced by seasonal variation (*p* < 0.001, Table 5). The EDI values of Fe, Zn, Cu, Ni, and Cr obtained from the muscle of studied specimens were compared to their toxicity reference values. These comparisons were made using the Reference Dose (RfD) and Provisional Maximum Tolerable Daily Intakes (PMTDI).

Health risk evaluation for iron, zinc, copper, nickel, and chromium in the muscle of sardine from the Moroccan Mediterranean coast^a^

**Table:** 

		Wn			Sp			Sm			Aut		
		EDI	THQ	*E*/*P*	EDI	THQ	*E*/*P*	EDI	THQ	*E*/*P*	EDI	THQ	*E*/*P*
Iron	Beni Ensar	33.46 ± 2.18	0.061 ± 0.00	0.05	24.17 ± 4.74	0.043 ± 0.000	0.03	16.32 ± 0.43	0.029 ± 0.000	0.02	22.29 ± 1.96	0.040 ± 0.000	0.03
	Ras el Ma	35.08 ± 0.09	0.063 ± 0.00	0.05	27.68 ± 0.90	0.050 ± 0.00	0.04	16.95 ± 2.77	0.030 ± 0.00	0.02	21.51 ± 0.80	0.039 ± 0.00	0.03
	El Houceima	35.06 ± 1.37	0.063 ± 0.00	0.05	22.33 ± 0.31	0.040 ± 0.00	0.03	17.62 ± 0.27	0.031 ± 0.00	0.03	24.48 ± 0.31	0.044 ± 0.00	0.03
Zinc	Beni Ensar	53.58 ± 7.33	0.17 ± 0.02	0.18	33.12 ± 6.70	0.11 ± 0.02	0.11	22.64 ± 2.01	0.08 ± 0.006	0.08	38.09 ± 6.95	0.13 ± 0.02	0.12
	Ras el Ma	67.30 ± 0.74	0.22 ± 0.002	0.22	29.31 ± 2.77	0.09 ± 0.009	0.10	25.98 ± 3.64	0.09 ± 0.01	0.09	36.08 ± 0.62	0.02 ± 0.002	0.12
	El Houceima	62.80 ± 0.55	0.21 ± 0.001	0.21	23.34 ± 0.59	0.08 ± 0.002	0.08	27.17 ± 1.08	0.08 ± 0.002	0.09	17.52 ± 0.29	0.06 ± 0.00	0.06
Copper	Beni Ensar	1.63 ± 0.10	0.04 ± 0.02	0.02	1.25 ± 0.08	0.03 ± 0.002	0.03	1.64 ± 0.5	0.04 ± 0.02	0.04	2.02 ± 0.8	0.05 ± 0.02	0.05
	Ras el Ma	0.64 ± 0,04	0.02 ± 0.00	0.02	1.22 ± 0.02	0.03 ± 0.000	0.03	0.79 ± 0.05	0.03 ± 0.001	0.02	0.65 ± 0.04	0.02 ± 0.001	0.02
	El Houceima	—	—	—	1.47 ± 0.02	0.04 ± 0.00	0.04	2.26 ± 0.01	0.06 ± 0.00	0.05	0.72 ± 0.006	0.02 ± 0.00	0.02
Nickel	Beni Ensar	—	—	—	—	—	—	0.51 ± 0.05	0.03 ± 0.002	—	0.65 ± 0.15	0.03 ± 0.007	0.03
	Ras el Ma	—	—	—	0.71 ± 0.02	0.03 ± 0.001	0.04	0.77 ± 0.34	0.04 ± 0.01	—	0.91 ± 0.007	0.046 ± 0.00	0.05
	El Houceima	0.25 ± 0.006	0.01 ± 0.00	0.01	—	—	—	0.72 ± 0.08	0.04 ± 0.004	0.04	0.61 ± 0.008	0.031 ± 0.00	0.04
TTHQ	Beni Ensar	0.29 ± 0.008			0.18 ± 0.03			0.17 ± 0.01			0.25 ± 0.007		
	Ras el Ma	0.31 ± 0.002			0.22 ± 0.01			0.18 ± 0.03			0.22 ± 0.002		
	El-Houceima	0.13 ± 0.00			0.16 ± 0.003			0.22 ± 0.02			0.30 ± 0.003		
CR_Ni_	Beni Ensar	ND			ND			1.02 × 10^−4^			1.2 × 10^−4^		
	Ras el Ma	ND			1.4 × 10^−4^			1.5 × 10^−4^			1.8 × 10^−4^		
	El Houceima	4.57 × 10^−5^			ND			1.32 × 10^−4^			1.12 × 10^−4^		

a The results are presented as mean ± standard deviation; Wn: winter; Sp: spring; Sm: summer; Aut: autumn; EWI (µg kg^−1^ body weight): estimated weekly intake; THQ: target hazard quotient; CR_Ni_: carcinogenic risk related to nickel; TTHQ: total target hazard quotient; THQ; PTDI: permissible tolerable daily intake in µg kg^−1^ body weight per day.^36^

**Table:** 

PTDI	Iron	Zinc	Copper	Nickel	Chromium
	700	300	40	20	3

**Table 5:** Analysis of variance regarding the effect of site, season, and their interaction on EDI, and THQ and THQ contamination levels in the sardine muscle from the Moroccan Mediterranean coasts^a^

	Degree of freedom	Mean squares	*F*	*P*
**EDI**_**Fe**_				
Site	2	4.809	1.40	>0.05
Season	3	481.152	140.644	<0.001
Site × season	6	9.480	2.771	<0.05
Error	24	3.421		

**EDI**_**Cu**_				
Site	2	1.951	7.753	<0.01
Season	3	0.997	3.964	<0.05
Site × season	6	1.167	4.638	<0.05
Error	24	0.252		

**EDI**_**Zn**_				
Site	2	147.176	10.085	<0.01
Season	3	2505.746	171.71	<0.001
Site × season	6	158.289	10.847	<0.001
Error	24	14.593		

**EDI**_**Ni**_				
Site	2	0.291	23.281	<0.001
Season	3	0.914	73.160	<0.001
Site × season		0.136	10.907	<0.001
Error	24	0.012		

**THQ**_**Fe**_				
Site	2	1.575^E^-005	1.406	>0.05
Season	3	0.002	140.644	<0.001
Site × season	6	3.10^E^-0.005	2.771	<0.05
Error	24	1.12^E^-0.005		

**THQ**_**Zn**_				
Site	2	0.002	10.085	<0.01
Season	3	0.028	171.71	<0.001
Site × season	6	0.002	10.847	<0.001
Error	24	0.000		

**THQ**_**Cu**_				
Site	2	0.001	7.753	<0.01
Season	3	0.001	3.964	<0.05
Site × season	6	0.001	4.638	<0.05
Error	24	0.000		

**THQ**_**Ni**_				
Site	2	0.001	23.281	<0.001
Season	3	0.002	73.160	<0.001
Site × season	6	0.000	10.907	<0.001
Error	24	3.122^E^-005		

**TTHQ**				
Site	2	0.002	10.085	<0.01
Season	3	0.002	73.160	0.000
Site × season	6	0.11	42.691	<0.001
Error	24	0.000		

a EWI (µg kg^−1^ body weight): estimated weekly intake; THQ: target hazard quotient; CR: carcinogenic risk; TTHQ: total target hazard quotient; THQ.

#### 3.2.1. Estimated daily intake

The highest EDI values were recorded during the cold season for Fe (35.08 µg kg^−1^ body^−1^ weight (BW) day^−1^) and Zn 67.30 µg kg^−1^ BW^−1^ day^−1^. Meanwhile, the higher values for Cu (12.26 µg kg^−1^ BW^−1^ day^−1^) and Ni (0.91 µg kg^−1^ BW^−1^ day^−1^) were recorded during the hot season. The calculated values were compared to the recommended limits of oral RfD preconized by the European Food Safety Authority for Fe, Zn, Cu, Ni and Cr are 700, 300, 40, 20, and 3 µg kg^−1^ BW day^−1^, respectively.^12–14,36^ As shown in Table 4, the EDI for all studied meals is below the RfD, reflecting low percentages of the reference values (ranging between 1% (EDI_Ni_) and 22% (EDI_Zn_) for a person weighing 73.35 kg). Based on these results, it can be concluded that the intake of these metals through sardine muscle from Mediterranean Moroccan coasts is unlikely to pose any risk to the average consumer. The recorded values are comparable to those found by^14^ for sardines from Bangladesh,^34^ for sardines from the Spanish Mediterranean, and by^35^ for sardines from China (Table 6). However, these values are lower than those recorded for canned sardines consumed in Iraq and Nigeria,^38,39^Table 5. This difference could certainly be linked to processing factors during canning, such as the incorporated ingredients (oil, tomatoes, and spices) and the drying conditions. The ratio of EWI/PTWI can be used to evaluate the human health risks related metal exposure through food consumption. The calculated *E*/*P* ratios are presented in Table 4. In this study, the *E*/*P* values for trace metals in sardine muscle across all locations and seasons were below 1, indicating low risk.

**Table 6:** Comparison of the health risk indices obtained for Fe, Zn, Cu, Ni, and, Cr in sardine muscle with the reported values in the literatures^a^

		Beni-Ensar, Morocco	Ras el Ma, Morocco	El-Houceima, Morocco	Brasil^33^	Yang, China^35^	Egypt^13^	Bangladesh^14^	Spain^34^	Nigeria^39^
		Current study	Current study	Current study						
EDI	Fe	16.62–33.46	16.95–35.08	17.62–35.06	—	0.91	2.39	—	—	71.79–430
	Zn	22.64–53.58	25.98–67.30	17.52–62.80	—	0.88	0.55	10–15	0.47	0.8–41.34
	Cu	2.02–1.25	0.64–1.22	0–12.26	—	0.016	0.06	2.5–7.5	0.12	0.09
	Ni	0–0.65	0–0.91	0–0.72	—	0.007	0.05	0.5	—	0.36–29.11
	Cr	ND	ND	ND	—	0.012	0.02	0.1	—	0.09–0.89
THQ	Fe	0.029–0.061	0.03–0.063	0.031–0.063	0.002–0.068	0.0015	0.003	—	—	—
	Zn	0.08–0.17	0.02–0.22	0.08–0.21	0.005–0.042	0.0030	0.00185	0.042821	—	—
	Cu	0.03–0.05	0.02–0.03	0–0.04	0.001–0.039	0.0005	0.00155	0.081492	—	—
	Ni	0–0.03	0–0.04	0.04		0.0004	0.00250	—	—	—
	Cr	ND	ND	ND	0.005–0.031	0.0040	0.000013	0.034222	—	—
TTHQ		0.17–0.29	0.18–0.31	0.13–0.30	0.460–2.44	0.4640	0.02195	1.266326	—	—

a Fe: iron; Zn: zinc; Cu: copper; Ni: nickel; Cr: chromium; The results are presented as mean ± standard deviation; EWI (µg kg^−1^ body weight): Estimated weekly intake; THQ: target hazard quotient; CR: carcinogenic risk; TTHQ: total target hazard quotient; THQ; PTDI: permissible tolerable daily intake in µg kg^−1^ body weight/day; ND: not detected.

#### 3.2.2. Non-carcinogenic risk assessment

The calculated THQ and TTHQ for individual metals through the consumption of different sites and seasons time are presented in Table 4. There's a notable seasonal influence on THQ values, with higher levels of Fe and Zn detected in colder seasons, *p* < 0.001, Table 4 (THQ_Fe_: 0.063; THQ_Zn_: 0.09). However, the differences across sampling locations aren't statistically significant (*p* > 0.05). All the estimated THQ values are below the permissible limit (1). Indicating that there is no significant health risk associated with consuming the sardine muscle with respect to the specific contaminant being evaluated. Several previous studies for sardine muscle have also recorded values below 1.^33,35^ We also calculated the TTHQ to evaluate the contamination risk posed by the five metals (Fe, Zn, Cu, Ni, and, Cr). The results obtained from the maximum safe consumption quantity index (data not shown) align with the THQ data, indicating no potential human health risks from consuming Moroccan Mediterranean sardines.

The TTHQ serves to assess the non-carcinogenic risk of food potentially contaminated by multiple chemical elements. It is determined by summing the Target Hazard Quotients (THQs) for the metals studied. Table 4 presents the TTHQ values for the five chemical elements. All TTHQ values are below 1, which implies that consuming sardines from the Mediterranean coast of Morocco is generally safe with respect to these metals. Several studies, including those by^33^ in Brazil,^35^ in China, and^14^ in Bangladesh, report a higher TTHQ than our findings. However, our results are higher than those reported by^13^ for sardine muscle from the Egyptian Mediterranean coast (Table 6).

#### 3.2.3. Potential carcinogenic risk

Toxicological evaluation using carcinogenic risk (CR) represents the increased likelihood of developing cancer over one's lifetime due to exposure to carcinogens.^40^ It is a key metric used to evaluate the potential for cancer development. The CR index was applied to the data obtained for the concentrations of Fe, Zn, Cu, Ni, and Cr in sardine samples. In the current study, CR values were assessed specifically for Ni Cu and Cr because these metals are known to carry carcinogenic risks. The Cancer slope factors for Ni and Cr are 0.91 and 0.5 mg kg^−1^ day^−1^, respectively. The recorded target cancer risk values for Ni exceed 10^−4^ (Table 4), indicating a potential cancer risk to human health. In contrast, the risk associated with Cr is absent across all locations and seasons, suggesting that long-term exposure of sardines is unlikely to cause carcinogenic effects from Cr ingestion, as Cr was not detected in any of the studied muscles.

### 3.3. Chemometric analysis

**Fig. 2:**
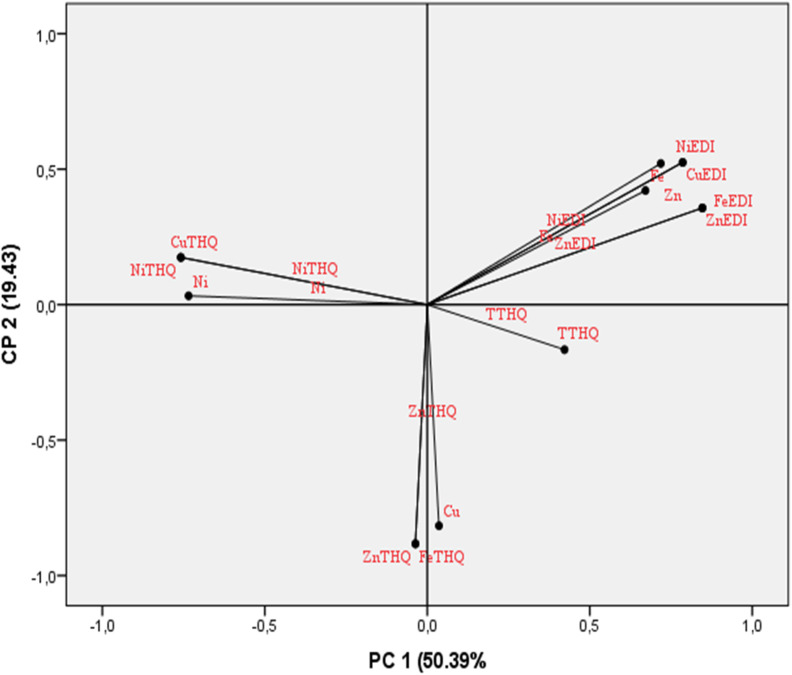
Projection of trace element concentrations, EDI, THQ, and TTHQ in the plane defined by two principal components. Fe: iron; Zn: zinc; Cu: copper; Ni: nickel; EDI: Estimated Daily Intake; THQ: Target Hazard Quotient, and TTHQ: Total Target Hazard Quotient.

A principal component analysis (PCA) was conducted to investigate and clarify the relationships between trace element accumulation in the edible muscle of Moroccan Mediterranean Sardines and their associated health risk indices. This approach provided deeper insights into the previously obtained results by analyzing 13 variables, including trace element concentrations, EDI, THQ, and TTHQ. The PCA offers a clear visualization of how these factors correlate with metal content and their corresponding health risk indices. Fig. 2 depicts the distribution of these variables within the plane defined by the first 2 principal components (PCs).

**Fig. 3:**
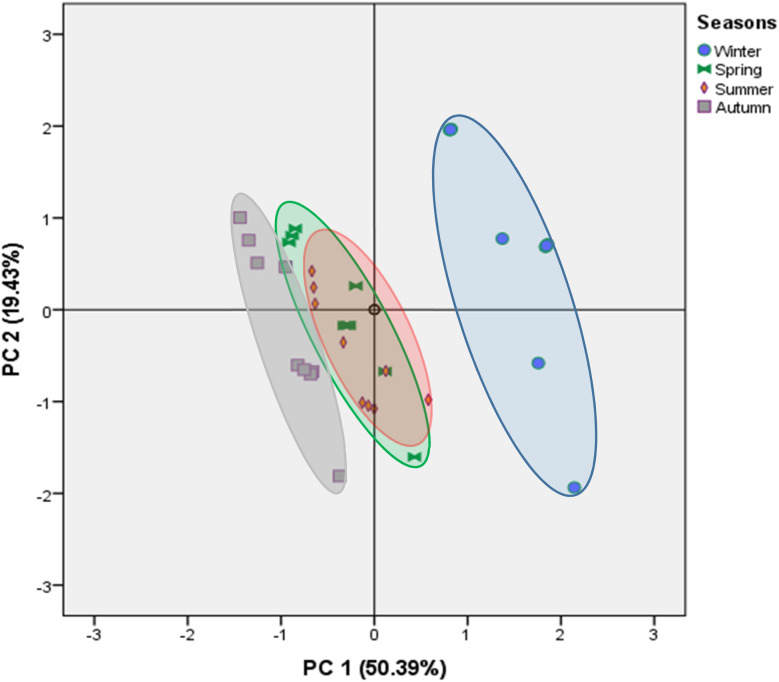
Projection of the variables of the four studied seasons in the plane defined by two principal components.

The three first PC represent 82.43% of the variation, accordingly (Table 7). The PC1 (50.39%) was correlated positively with Fe, Zn, EDI_Fe_, and EDI_Zn_, and negatively with Ni content and THQ for Ni and Cu. The PC2 (19.43%) was characterized negatively by THQ_Zn_, THQ_Fe_, and Cu concentration sardine muscle. The projection of the analyzed specimens onto the plane defined by the first two PCs reveals a clear seasonal variation in contamination levels (Fig. 3). Regardless of the geographical area, specimens from the winter season are positioned on the right side of Fig. 3, distinguished by higher concentrations of Fe and Zn (Fig. 2). In contrast, those from the autumn season appear on the left side of Fig. 3, characterized by greater levels of Ni and Cu (Fig. 2). This observed distinction among the sardines under study indicates a significant variation likely linked to the time of sampling (season). Our findings align with existing literature on contamination levels in marine ecosystems.^14,31,32^ The correlation matrix analysis indicates a positive relationship between Fe and Zn (*r* = 0.82) and a negative relationship between Fe and Ni (*r* = −0.53).

**Table 7:** Three main components explain more than 82.43% of the total information on contamination levels in sardine muscle from the Moroccan Mediterranean coasts^a^

Variables	Principal component			Variable	Principal component		
	1	2	3		1	2	3
Fe	0.792	—	0.320	EDI_Ni_	0.944	—	—
Zn	0.884	—	—	THQ_Fe_	−0.481	0.741	0.225
Cu	−0.384	0.721	−0.162	THQ_Zn_	−0.481	0.741	0.225
Ni	−0.616	−0.402	−0.411	THQ_Cu_	−0.564	−0.536	0.595
EDI_Fe_	0.910	0.124	—	THQ_Ni_	0.910	0.124	—
EDI_Zn_	0.910	0.124	—	TTHQ	0.279	0.358	0.723
EDI_Cu_	0.944	—	—				

a Fe: iron; Zn: zinc; Cu: copper; Ni: nickel; EDI: Estimated Daily Intake; THQ: Target Hazard Quotient, and TTHQ: Total Target Hazard Quotient.

## 4. Conclusion

This research highlights the dual role of trace elements in supporting physiological functions while posing potential health risks when their concentrations exceed recommended levels. The analysis of *Sardina pilchardus* muscle from the Moroccan Mediterranean coast revealed significant seasonal variations in metal concentrations, with zinc (Zn) and iron (Fe) being the most prevalent, and nickel (Ni) showing the lowest levels. Despite these variations, the concentrations of Zn, Fe, and copper (Cu) were within international food safety limits, indicating no immediate health risks from consuming these fish. The health risk assessment based on indices such as EDI, THQ, and total THQ confirms that consuming *S. pilchardus* generally poses no immediate health risk. However, the potential long-term carcinogenic risk associated with nickel, whose levels exceeded safety thresholds, underscores the need for continuous monitoring, given these metals' toxicological impact and environmental persistence. In conclusion, while *S. pilchardus* remains a nutritious food source, the potential health risks related to trace element contamination, particularly nickel, warrant careful consideration and ongoing surveillance to ensure consumer safety. In conclusion, while *S. pilchardus* remains a nutritious food source, the potential health risks related to trace element contamination, particularly nickel, warrant careful consideration and ongoing surveillance to ensure consumer safety. This study has some limitations, including the restricted number of sampling sites and the focus on muscle tissue only, which may not fully reflect the overall bioaccumulation within the species. Moreover, the study considered a limited range of metals, whereas including other contaminants such as Pb, Cd, and Hg would provide a more comprehensive assessment. Future research should therefore aim to broaden the sampling area and period, include additional organs and contaminants, and explore the influence of environmental factors on metal accumulation. In addition, emerging pollutants such as aluminum, microplastics, per- and polyfluoroalkyl substances (PFAS), bisphenol A (BPA), and organic contaminants (PAHs, PCBs, pesticides) should be investigated as they increasingly interact with trace metals and may influence their bioavailability and toxicity. Integrating these parameters with biomarker and isotopic approaches could provide deeper insights into contamination pathways and their combined effects on both ecosystem and human health.

## Author contributions

All authors contributed to the study conception and design. Material preparation, data collection and analysis were performed by Khaoula Kasmi, Douaa Slimani, Mouedden Rajae and Hanae Nasri. The first draft of the manuscript was written by Khaoula Kasmi and kamal belhaj and all authors commented on previous versions of the manuscript. All authors read and approved the final manuscript.

## Conflicts of interest

The authors declare that they have no competing interests.

## Data Availability

The data used to support the findings of this study are included within the article.
